# Management and Prognosis of Patients with Recurrent or Persistent/Progressive Uterine Carcinosarcoma

**DOI:** 10.3390/curroncol29100601

**Published:** 2022-10-13

**Authors:** Hsiu-Jung Tung, Chi-Yuan Chiang, Wei-Yang Chang, Ren-Chin Wu, Huei-Jean Huang, Lan-Yan Yang, Chiao-Yun Lin, Chun-Chieh Wang, Angel Chao, Chyong-Huey Lai

**Affiliations:** 1 Department of Obstetrics and Gynecology, Chang Gung Memorial Hospital, Linkou Branch and Chang Gung University College of Medicine, Taoyuan 333, Taiwan; 2 Gynecologic Cancer Research Center, Chang Gung Memorial Hospital, Taoyuan 333, Taiwan; 3 Clinical Trial Center, Chang Gung Memorial Hospital, Taoyuan 333, Taiwan; 4 Department of Pathology, Chang Gung Memorial Hospital, Linkou Branch and Chang Gung University College of Medicine, Taoyuan 333, Taiwan; 5 Department of Radiation Oncology, Chang Gung Memorial Hospital, Linkou Branch and Chang Gung University College of Medicine, Taoyuan 333, Taiwan

**Keywords:** carcinosarcoma, MMMT, endometrial cancer, survival after recurrence

## Abstract

Uterine carcinosarcoma (UCS) is a highly aggressive gynecologic malignancy. Recurrent or persistent/progressive disease is usually fatal. We aimed to investigate the management and prognosis of these patients. Clinical records of UCS patients from June 1987 to April 2020 were retrospectively reviewed. The stage was re-assigned with the FIGO 2009 staging system. Univariate and multivariate analyses were used to identify the independent predictors of survival after recurrence (SAR) and cancer-specific survival (CSS). Of the 168 patients, 98 experienced treatment failure. The median time to treatment failure (TTF) was 8.1 months (range: 0.0–89.1). The median follow-up time of censored patients was 32.0 months (range: 16.8–170.7). The 5-year SAR rates of those with recurrent or persistent/progressive disease were 7.6%. On multivariate analysis, salvage therapy mainly using radiotherapy (HR 0.27, 95% CI: 0.10–0.71) or chemotherapy (HR 0.41, 95% CI: 0.24–0.72) or chemoradiotherapy (CRT) (HR 0.33, 95% CI: 0.15–0.75) were associated with improved SAR, whereas disseminated recurrence was associated with significantly worse SAR (HR 3.94, 95% CI: 1.67–9.31, *p* = 0.002). Salvage therapy using radiotherapy or chemotherapy or CRT significantly improved SAR. Surgery significantly improved CSS but not SAR, adjusting for confounding factors.

## 1. Introduction

Uterine carcinosarcoma (UCS) is a rare endometrial cancer that has both epithelial and mesenchymal malignancy cell components, also named malignant mixed Mullerian tumor (MMMT). The incidence in previous literature was less than 5% of primary endometrial cancer [1] but now exceeds 5%, with a continuously increasing trend, from 1.7% to 5.6% between 1973 and 2013 [2]. An increasing annual percent change in UCS incidence from 2001–2017 was also observed in the United States [3]. UCS is notorious for a poorer prognosis than other types of endometrial carcinoma [4,5]. Unlike endometrioid adenocarcinoma, which only presents with extrauterine disease in 20.8% of cases, UCS presents with extrauterine disease in 56.3% of cases (*p* < 0.001), according to a study using the Surveillance Epidemiology and End Result database (1973–2010) [5]. There was significantly shorter 5-year overall survival (OS) for UCS patients: 14% as compared to 62% for the endometrioid adenocarcinoma patients [5].

UCS used to be categorized into sarcoma, as it has both a sarcomatous (mesenchymal) and a carcinomatous (epithelial) component [6]. It is now believed to be originated from a single endometrial tumor clone, which then underwent metaplastic differentiation (conversion hypothesis), rather than stemming from a simply “biphasic” tumor [7]. The epithelial part is usually poorly differentiated and heterogeneous with an endometrioid, clear-cell, or serous feature, and the mesenchymal part can be either homologous (endometrial stromal sarcoma or leiomyosarcoma) or heterologous (rhabdomyosarcoma, chondrosarcoma, osteosarcoma, and liposarcoma). However, these histologic features seem not to be related to prognosis [8]. When metastasis and lymphatic spread are detected, the carcinomatous component plays an important role. Comprising not only the majority of metastatic histology, the poorer prognosis also correlates to higher grade epithelial elements such as serous or clear-cell histology [9].

The primary treatment for UCS is surgical debulking, followed by adjuvant therapy including both chemotherapy and radiotherapy [10]. Even after receiving such aggressive treatment, high recurrence rates were encountered in 37%, 46%, 63%, and 80% of stages I, II, III, and IV patients, respectively [1]. Risk factors associated with recurrence included the International Federation of Gynecology and Obstetrics (FIGO) stage, depth of myometrial invasion, lymphovascular space invasion (LVSI), adnexa and serosal involvement, positive cytology, and lymph node metastasis [8,11]. Although adjuvant chemoradiotherapy (CRT) improves survival [8,12,13], there has been little information focused on recurrence patterns or salvage treatments in relation to outcome for those who experience treatment failure, as patients with recurrent/progressive endometrial cancer have few options for second-line therapies [14], not to mention those with UCS. A comprehensive investigation for molecular features in UCS should therefore be undertaken.

We aimed to explore those patients with recurrent or persistent/progressive disease and to focus on their salvage treatment strategies, including experiences in immunotherapy and target agents.

## 2. Materials and Methods

### 2.1. Patients

We retrospectively collected clinical information from the medical records of patients who were diagnosed with UCS in Chang Gung Memorial Hospital, Linkou Branch from June 1987 to April 2020 (Supplementary Figure S1). Informed consent was waived under institutional review board approval (IRB No.: 202101235B0). The histological diagnosis was made according to the World Health Organization’s classification by board-certified pathologists. We also had an independent gynecologic pathologist (R.C.W.) review all patients’ tissue slides to confirm the diagnosis according to the most recent diagnostic criteria. Patients who were eligible for analysis were followed until September 2021.

### 2.2. Primary Therapy and Adjuvant Choice

Patients were treated with primary surgery except for poor surgical candidates or those with multiple distant metastases. Neoadjuvant therapy or palliative radiotherapy was arranged if they could not receive primary surgery. Primary surgical staging includes peritoneal washing cytology, hysterectomy with bilateral salpingo-oophorectomy, and pelvic lymphadenectomy. Para-aortic lymph node (PALN) dissection was recommended but not mandatory. Disease staging was assigned according to FIGO 2009 classification [15]. For those who could not receive staging surgery, their staging would be defined with the available clinical information, such as image scans. After primary surgery, adjuvant treatments including chemotherapy, radiotherapy, or CRT were planned [8,10]. Only stage IA patients without myometrial invasion could forgo adjuvant therapy. We followed up on tumor markers and image scans (computed tomography (CT)/magnetic resonance imaging (MRI) or positron emission tomography/CT (PET/CT)) every 3–6 months during active treatment and every 6–12 months during surveillance [8].

### 2.3. Recurrent Patterns

CT- or ultrasound-guided biopsy/aspiration or thoracoscopic resection was performed to confirm the diagnosis of suspicious lesions detected by image scans. When suspicious lesions were detected by one image modality but guided biopsy was not feasible, a repeated evaluation using another image method was performed. The recurrent patterns were classified as isolated or disseminated. Isolated recurrence was defined as a single organ or area that could be resected or covered with radiotherapy, such as resectable/unradiated vaginal/pelvic recurrence or a solitary lung nodule. Disseminated recurrence was defined as multiple organs or areas of involvement.

### 2.4. Statistics

Patient characteristics were summarized by descriptive statistics. Intergroup comparisons between patients with isolated and disseminated diseases were performed using the Mann–Whitney U test (for continuous variables) and Fisher’s exact tests for categorical variables. Bivariate associations were examined by calculating the phi coefficient. Survival after recurrence (SAR) was measured from progression to death because of UCS, or it was censored at the last follow-up. Cancer-specific survival (CSS) was calculated from diagnosis to cancer-related death, and deaths because of intercurrent disease were censored. Kaplan–Meier curves were used to estimate survival rates. The differences in survival curves were compared by log-rank tests. Multivariate stepwise Cox proportional hazard regression models were used to identify the independent predictors of survival. Results were expressed as hazard ratios (HRs) with 95% confidence intervals (CIs). All statistical calculations were performed using the IBM SPSS statistical package (version 26.0; IBM Inc., Armonk, NY, USA). Two-tailed *p* values < 0.05 were considered statistically significant.

## 3. Results

### 3.1. Patients Characteristics and Survival

**Figure 1:**
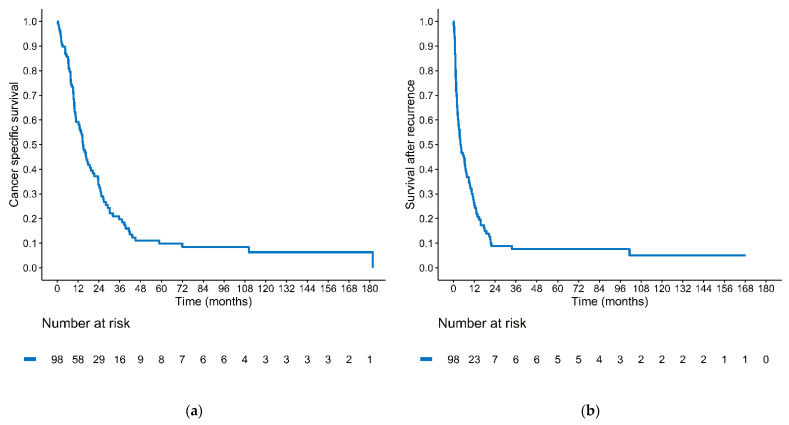
Kaplan–Meier estimates of (**a**) cancer-specific survival and (**b**) survival after recurrence in recurrent/progressive patients. (*N* = 98).

There were 191 patients diagnosed with UCS. We excluded those who did not receive treatment (*n* = 16), those who received a wrong diagnosis (*n* = 6), and those whose initial pathologic report could not be retrieved (*n* = 1). There were 168 patients eligible for analysis, and 69 patients did not have disease recurrence. One patient received surgery and decided to have further treatment in the United States (Supplementary Figure S1). There were 98 patients who experienced treatment failure. The median time to treatment failure (TTF) was 8.1 months (range: 0.0–89.1). The median follow-up time of censored patients was 32.0 months (range: 16.8–170.7). The 5-year CSS rate of these recurrent or persistent/progressive UCS patients was 9.8% (Figure 1a). The 5-year SAR rate was 7.6%, with a median of 3.9 months (range: 0.1–168.2 months) (Figure 1b).

Of these 98 patients, only 14 (14.3%) patients had an isolated recurrence, and 84 (85.7%) had disseminated recurrences. Table 1 depicts demographic characteristics of the recurrent/progressive UCS patients. The disseminated group had significantly shorter TTF than the isolated group (7.4 (range: 0.0–68.5) vs. 16.2 (range: 6.6–89.1) months, *p* = 0.001) and higher CA-125 levels at recurrence. However, between these two groups, there was no difference in age distribution, estrogen receptors (ER), progesterone receptors (PR), primary treatment, postoperative adjuvant treatment, salvage target and immune therapy, and radiotherapy failure type. Salvage therapies are summarized in Table 2. More patients in the disseminated group did not receive salvage treatment but received best supportive care (40.7% vs. 7.7%, *p* = 0.002), as compared with the isolated group. In those who received chemotherapy (*n* = 36), paclitaxel and platinum (*n* = 10, 10.6%) were the most commonly chosen therapies, followed by anthracycline and platinium agents (*n* = 7, 7.4% and *n* = 7, 7.4%). Novel target/immune agents were tried at first-line salvage therapies in 9 (9.6%) patients.

### 3.2. Factors Influencing CSS and SAR

To investigate CSS, univariable and multivariable analyses were performed. Patients who had a treatment failure time of >6 months had more favorable outcomes (HR 0.30, 95% CI: 0.18–0.52, *p* < 0.001) than those whose illnesses recurred/progressed shortly (TTF ≤ 6 months). Disseminated recurrence resulted in poor prognosis than isolated recurrence (HR 4.01, 95% CI: 1.55–10.33, *p* = 0.004). All types of salvage treatments prolonged CSS except HT (surgery ± chemotherapy or radiotherapy or CRT or target: HR 0.44, *p* = 0.033; chemotherapy ± HT/target therapy: HR 0.29, *p* < 0.001), especially CRT ± HT/target therapy (HR 0.17, 95% CI: 0.11–1.01, *p* < 0.001) compared with supportive care by multivariable analysis. HT alone was associated with significantly improved CSS in the univariate analysis but was marginal (*p* = 0.053) in the multivariate analysis (Table 3).

In the multivariate analysis for SAR, the most important factor was a recurrent pattern (disseminated vs isolated: HR 3.94, 95% CI: 1.67–9.31, *p* = 0.002). The SAR curves sorted by recurrent pattern are illustrated in Supplementary Figure S2. Most types of salvage modalities improved SAR (chemotherapy (HR 0.41, 95% CI: 0.24–0.72, *p* = 0.002), radiotherapy (HR 0.27, 95% CI: 0.10–0.71, *p* = 0.008), CRT (HR 0.33, 95% CI: 0.15–0.75, *p* = 0.008)) compared with supportive care by multivariable analysis, whereas HT alone was associated with significantly improved SAR in the univariate but not the multivariate analysis (Table 4). In the disseminated group, the most relevant prognostic factor for SAR was salvage treatment. Patients receiving chemotherapy, radiotherapy, and CRT still had better SAR. Surgery and HT did not prolong SAR (Supplementary Table S1). In the isolated group, the most relevant prognostic factor for SAR was age > 70 years (median SAR 11.1 vs. 33.6 months, *p* < 0.001) (Supplementary Table S2). Obviously, patients of age > 70 could not tolerate aggressive salvage treatment even if it might be potentially curative, and surgery alone +/− HT or target therapy marginally improved SAR (5-year SAR 100% vs. 27.5%, *p* = 0.072).

### 3.3. Long-Term Survivors

Of the stage I/II patients with treatment failure (*n* = 26), three patients (Patients 1, 2, and 5 in Table 5) were successfully salvaged. Salvage surgery or radiotherapy/CRT or target therapy was associated with significantly better SAR than chemotherapy alone ±HT/target therapy or HT alone (Supplementary Figure S3A). Patient 1 had stage IA cancer with myometrial invasion of 20%. She chose to undergo surgery alone without adjuvant treatment. Disseminated recurrences were salvaged with CRT to the pelvis and lung, and she experienced complete remission for 168.2 months. Patient 2 had stage II disease that recurred with solitary lung metastasis and was cured with lung resection. Patient 5 with stage IA disease had a solitary vaginal recurrence and remained free from evidence of disease after a partial vaginectomy and vaginal radiotherapy.

Among those who had initial stage III/IV disease (*n* = 72), 60 (83.3%) had a disseminated recurrence, and only one of these had a disease control for the first relapse. The patient had a stage IVB disease with peritoneal carcinomatosis. She received sandwich CRT after primary surgery but had subdiaphragm and vaginal recurrence. Radiotherapy and megestrol acetate were tried, and complete metabolic remission was noted on a PET scan. However, the patient died of pulmonary emboli shortly after her disease remission (SAR of 20.5 months). Nevertheless, 3 of 13 patients with a solitary relapse were cured (Patients 4 and 6) or experienced long-term remission (Patient 3). Patient 3 was in stage IIIB with a deep infiltrated parametrium to the left pelvic side wall. After postoperative CRT, she failed in the previously irradiated field (left pelvic side wall). HT with anastrozole and megestrol acetate was given. Her lesion finally received metabolic complete remission (by MRI and PET/CT). Re-recurrence occurred after she discontinued the HT, and she died despite further immune/target therapy. Patient 4, with stage IIIC2 disease, had a solitary recurrence over a neck LN and was successfully salvaged with radiotherapy. Patient 6, with stage IIIA disease, had a solitary lung recurrence and a complete response to radiotherapy. The SAR curves of different salvage modalities for stage III-IV patients are illustrated in Supplementary Figure S3B.

### 3.4. Molecular Medicine

Due to the salvage chemotherapy variety, it is not feasible to analyze all regimens. In the 36 patients who received salvage chemotherapy, regimens containing ifosfamide had a better SAR than those that did not (*p* = 0.041, Supplementary Figure S4). On the other hand, chemotherapy containing paclitaxel or platinum was not related to better SAR (Supplementary Figure S5).

Novel therapies included in the first salvage plan were also analyzed. There were nine patients whose salvage modalities including target or immunotherapy (Table 2). The salvage treatment plan of these patients is illustrated in Table 6. Patient 1 received RT to a left inguinal LN recurrence and achieved CR, then maintained with Olaparib (her tumor had BRCA1 deletion). However, Olaparib was interrupted shortly thereafter due to pancytopenia. She had a re-recurrence 12 months later and was tried on Pembrolizumab, but died of sepsis and intractable thrombocytopenia. There were four patients presenting as stage IVB disease initially. Eight patients had a disseminated recurrence. Their SAR curve compared with the no novel therapies group was not significantly different (Supplementary Figure S6).

## 4. Discussion

Although much literature has explored prognostic factors [8,11,13,16] and adjuvant treatments [8,16,17,18,19], reports focusing on treatment failure of UCS are scarce. In our study, patients with recurrent or persistent/progressive UCS experienced treatment failure with a median TTF of 8.1 months (range: 0.0–89.1). The 5-year SAR rates were 7.6% (median: 3.9 months, range: 0.1–168.2). TTF ≤ 6 months was independent of adverse prognostic factors for CSS but not for SAR. Disseminated recurrence significantly impacted CSS and SAR. Salvage therapy consisting of radiotherapy or chemotherapy or CRT significantly improved CSS and SAR. Surgery significantly improved CSS but not SAR, adjusting for confounding factors.

Adjuvant CRT after surgery has been known to prolong survival even in early stages [8,17,19], especially with paclitaxel plus carboplatin [20]. However, there was no consensus about second-line salvage treatment when first-line chemotherapy failed. A report analyzed 42 patients who received second-line chemotherapy with disappointing efficacy and found a poor PFS and OS [21]. Matsuo et al. reported a better two-year SAR with taxane/platinum; however, 69% (49/71) of patients who received taxane/platinum were chemotherapy- or taxane-/platinum-naïve [22]. In our series, regimens containing taxane or platinum were not beneficial, whereas regimens containing ifosfamide resulted in a better SAR (*p* = 0.041) than other regimens among the 36 patients who received salvage chemotherapy, and only 22.2% (8/36) of them were chemotherapy-naïve. Our results were consistent with another Japanese case series using ifosfamide and paclitaxel that revealed a great overall response rate (ORR) of 38.4% and a disease control rate of 92.3%. The toxicity was also manageable without treatment-related death [23].

As UCS belongs to endometrial cancer, around two-thirds of cases had an endometrioid carcinoma component [7]. Hormone therapy has been discussed in UCS. A case series (*n* = 11) explored ER/PR in UCS and found 36.4% had one or both receptors [24]. Our available ER/PR expressions were 33.8% and 36.4%, respectively. Although consistent to this case series, our positive rate of ER/PR was relatively high compared to a recent article [25]. The positive rate of ER/PR may be affected by (1) the fraction of the carcinoma component in the tissue block chosen for immunostaining [26] and (2) the cutoff for positivity of ER/PR. Currently, there is no consensus on the cutoff for endometrial cancer. Following the ASCO/CAP guidelines for breast cancer, we have chosen one percent as the cutoff for endometrial cancer, which may have resulted in a higher fraction of ER/PR in our study. A case report demonstrated that a stage IIIC2 UCS patient with a solitary PALN recurrence had achieved long-term survival (SAR > 40 months) using megace, letrozole, and steostatic RT to PALN [27]. The PARAGON trial (ANZGOG 0903) evaluated anastrozole in ER- and/or PR-positive leiomyosarcoma and UCS. For the UCS cohort, the clinical benefit at 3 months was 43%, with a median duration of clinical benefit of 5.6 months [28]. An extraordinary outcome was also seen in our Patient 3 (Table 5), who was also in stage IIIB condition with infield failure but was successfully controlled with anatrozole and megestrol acetate, with a SAR of 101.4 months. However, HT alone was associated with significantly improved SAR in a univariate but not multivariate analysis in our series.

In the era of precision medicine, molecular subtypes have been explored for UCS. A genomic characterization of UCS from 57 patients was performed using exome sequencing, which showed a prominent *TP53* mutation (91%). The PI3-kinase pathway genes, including *PIK3CA* (35%), *PTEN* (19%), or *PIK3R1* (11%), also account for nearly half of these patients. Other significant mutated genes were *FBXW7* (28%), *PPP2R1A* (28%), *CDH4* (18%), *KRAS* (12%), *ARID1A* (12%), *ARHGAP35* (11%), *SPOP* (7%), and *RB1* (11%) [29]. In a pooled analysis of four studies using the the Cancer Genome Atlas (TCGA)’s molecular classifications, UCS had more copy-number-high (CNH) (73.9%) cases than copy-number-low (CNL) (13.5%) cases, with similar proportions of mutations in the exonuclease domain of DNA polymerase ε (POLE) (5.3%) and microsatellite instability (7.3%) [30]. Travaglino et al. performed a systemic review to compare TCGA grouping between UCS and the POLE mutation. UCS had an excellent outcome (*n* = 12, no recurrence, no death). Using a TP53mutation/p53abnormal/copy-number-high group as a reference, the MMRd group hazard ratio for progression-free survival (PFS) of UCS was: 0.19, 95% confidence interval (CI) 0.08–0.46, *p* < 0.001; whereas NSMP HR was 1.02, 95% CI 0.59–0.78, *p* = 0.936; and HRs for OS were not significant (0.91 and 1.51; *p* = 0.788 and 0.240) [31]. However, due to the majority of UCS falling into the TP53mutation/p53abnormal/copy-number-high group [32], the aggressive behavior creates a clinical dilemma, and recurrences are usually of a catastrophic condition.

Immune check point inhibitors (ICIs) have been tested in endometrial cancer. Programmed death-1(PD-1) antibodies (pembrolizumab and dostarlimab) have been approved by the US FDA and EMA for mismatch repair-deficient (MMRd) endometrial cancer. However, MMRd in UCS was rare, with only 4 (4/103) to 6.2% (16/276) being detected [33,34]. Other predictive biomarkers for using ICIs, programmed death-ligand 1(PD-L1), and PD-L2 were detected in 25% of the 59 cases with UCS [35]. A Japanese case report with a stage IB UCS (PD-L1 focally positive, microsatellite instability+) showed a relapse one month after primary surgery and resistance to first-line salvage chemotherapy, but an achievement of complete response with pembrolizumab and radiotherapy and survival for 14 months after pembrolizumab therapy [36]. Treatment response was also observed in other carcinosarcoma cases even with negative PD-L1 [36,37], yet the precise markers for the PD-1 antibody need to be determined. Another case that harbored missense mutations of the exonuclease domain of POLE (P286R and T323A), *PIK3CA*, *ARID1A*, and *PTEN* and a high tumor mutation burden (169 mutations per DNA megabase) achieved a durable response with pembrolizumab but was microsatellite stable [38]. The regimen of pembrolizumab and lenvatinib showed a more impressive outcome (HR for progression or death, 0.60, 95% CI: 0.50–0.72, *p* < 0.001) than chemotherapy in KEYNOTE-775 for metastatic and recurrent endometrial cancer [39]. Unfortunately, no UCS was included in KEYNOTE-775. Hunt et al. presented a small retrospective series with pembrolizumab and lenvatinib in seven patients of advanced or recurrent UCS as ≥ third-line, which observed no partial or complete response [40], while it was unknown if used earlier at first relapse. A ROCSAN trial with an anti-PD1 (dostarlimab) and a PARPi (niraparib) is now recruiting [41]. Of the four cases in our series that used pembrolizumab, two patients (Table 6, Patient 2, 6) with durable anti-tumor effects were observed, with 21.4 and 13.1 months SAR. These two patients were in a relatively early stage at diagnosis (stage IA with malignant ascites and stage IB). However, the other two cases with IVB disease (Table 6, Patients 1, 5) resulted in a short SAR after pembrolizumab.

Bevacizumab was used in several phase II trials for advanced/recurrent endometrial cancer. Likewise, those trials usually included no or very few UCS cases and had no significant PFS or OS benefit when added onto chemotherapy [42,43]. We had three cases using bevacizumab on recurrence; one of these using bevacizumab combined with cisplatin and pembrolizumab achieved 21.4 months SAR (Table 6, Patient 2). In a phase II study of recombinant fusion protein of human IgG1 with the principal cellular ligand-binding domains and the vascular endothelial growth factor receptor (VEGFR) 1 and 2 using Aflibercept as a single agent in patients with metastatic or recurrent gynecologic carcinosarcoma and uterine leiomyosarcoma, an ORR of 0% and 9% stable disease was seen in the carcinosarcoma cohort (19 uterine, 3 ovarian) [44]. Another small molecule inhibitor of VEGFR, pazopanib was tested in a phase II trial for recurrent/persistent UCS as a second- or third-line treatment, and no partial or complete response was seen [45]. Nishikawa et al. reported a case series of UCS (*n* = 7) or ovarian CS (*n* = 1) cancer, in which pazopanib provided a clinical benefit rate of 42.9% in UCS patients and a median PFS of 2.8 months (range: 0.8 to 11 months) [46]. Four patients treated with pazopanib in our series yielded 6.3 to 13.1 months SAR (Table 6, Patients 6–9).

DNA repair deficiencies in different cancer types have been proved to be a drug target. Tumors with homologous-recombination-deficiency (HRD) showed a high response to poly (ADP-ribose) polymerase (PARP) inhibitor [30]. A heavily treated ovarian carcinosarcoma (OCS) was found to respond to a PARP inhibitor with a *RAD51D* mutation [47]. Thus, a preclinical study using UCS and OCS cell lines with an HRD signature showed significantly more sensitivity to olaparib in vitro and in vivo when compared to homologous-recombination-proficiency (HRP) CS [48]. We had one case (Table 6, Patient 1) using olaparib combined with pembrolizumab and radiotherapy after neoadjuvant chemotherapy, extended field RT, supraclavicular LN RT, and adjuvant chemotherapy. She had an isolated relapsed at the left inguinal LN; however, she died from irreversible thrombocytopenia from bone marrow failure.

HER2 overexpression in UCS has also been studied recently, and ORRs were around 10–20% in UCS [6,49,50]. Although there is no clinical trial data using HER2-targeting agents in treating UCS, a phase II trial revealed that a trastuzumab add-on to carboplatin and paclitaxel prolonged PFS more than chemotherapy alone in stage III/IV HER2/neu over-expressed serous endometrial cancer [51]. Since these HER2-overexpressed UCS usually had a serous histology [49,50], HER2-targeted therapy would be a reasonable option in UCS with a serous component. We also had some patients with a check of HER2 IHC stain (*n* = 10/98). Eight of those patients were HER2 negative, and two of them were positive. However, they did not try Herceptin.

Although our experiences were limited in molecular-related target therapy, using next-generation sequencing (NGS) to explore druggable mutations has a great potential to guide future precise medicine. An analysis using NGS in 299 patients with gynecologic cancer revealed an improvement in average survival when target therapy was applied to actionable alterations [52]. That is, novel therapy must be used upfront for high-risk UCS; however, continued trials on novel therapies are the key to win the battle against recurrent/progressive UCS.

## 5. Conclusions

To date, there has been scant literature on optimal treatments for recurrent/progressive UCS. Conventional chemotherapy, although not curative, could prolong life. Surgery or radiotherapy can control small solitary tumors, whereas CRT should be considered for limited non-solitary failures in unradiated pelvises and on solitary distant sites. HT might be worth trying in patients with ER-/PR-positive tumors. Novel approaches must be used upfront for high-risk UCS defined by molecular classification. However, continued research on novel therapies is the key to improving the prognosis of such an aggressive disease.

## Figures and Tables

**Table 1:** Demographic characteristics of recurrent/persistent uterine carcinosarcoma patients.

Variable	Total (*n* = 98)		Isolated (*n* = 14)		Disseminated (*n* = 84)		*p*-Value ^1^
	*n*	%	*n*	%	*n*	%	
Age, years (median, range)	60.0 (32.3, 86.2)		58.6 (34.6, 80.3)		60.0 (32.3, 86.2)		0.768
CA-125, U/mL (median, range) at recurrence	49.8 (5.5, 3533.3)		19.9 (6.0, 136.7)		65.0 (5.5, 3533.3)		0.026
Time to treatment failure, months (median, range)	8.1 (0.0, 89.1)		16.2 (6.6, 89.1)		7.4 (0.0, 68.5)		0.001
Stage							0.509
I	20	(20.4)	3	(21.4)	17	(20.2)	
II	6	(6.1)	2	(14.3)	4	(4.8)	
III	35	(35.7)	5	(35.7)	30	(35.7)	
IV	37	(37.8)	4	(28.6)	33	(39.3)	
Estrogen receptor							0.354
Negative	49	(63.6)	10	(76.9)	39	(60.9)	
Positive	28	(36.4)	3	(23.1)	25	(39.1)	
Progesterone receptor							0.752
Negative	51	(66.2)	8	(61.5)	43	(67.2)	
Positive	26	(33.8)	5	(38.5)	21	(32.8)	
Primary treatment							0.702
Surgery	85	(86.7)	12	(85.7)	73	(86.9)	
Neoadjuvant chemotherapy	8	(8.3)	2	(14.3)	6	(7.1)	
Chemotherapy	2	(2.0)	0	(0)	2	(2.4)	
Radiotherapy	1	(1.0)	0	(0)	1	(1.2)	
Chemoradiation	1	(1.0)	0	(0)	1	(1.2)	
Hormone therapy	1	(1.0)	0	(0)	1	(1.2)	
Adjuvant treatment							0.434
No	18	(18.4)	2	(14.3)	16	(19.0)	
Radiotherapy	7	(7.1)	0	(0)	7	(8.3)	
Chemotherapy + radiotherapy	40	(40.8)	9	(64.3)	31	(36.9)	
Chemotherapy	32	(32.7)	3	(21.4)	29	(34.5)	
Hormone therapy	1	(1.0)	0	(0)	1	(1.2)	
Adjuvant chemotherapy regimen							0.560
No chemotherapy	27	(27.6)	2	(14.3)	25	(29.8)	
Platinum + Paclitaxel	32	(32.7)	5	(35.7)	27	(32.1)	
Platinum + Ifosfamide	13	(13.3)	3	(21.4)	10	(11.9)	
PAC or PC/PAI/PA	13	(13.3)	1	(7.1)	12	(14.3)	
Adrimycin + Ifosfamide	5	(5.1)	1	(7.1)	4	(4.8)	
Other	8	(8.2)	2	(14.3)	6	(7.1)	
Radiotherapy field failure							0.728
Non-infield failure	77	(78.6)	12	(85.7)	65	(77.4)	
Infield failure	21	(21.4)	2	(14.3)	19	(22.6)	

^1^ Comparing isolated and disseminated recurrence using Fisher’s exact test for categorical variables or Mann–Whitney *U* test for continuous variables. Abbreviations: PAC, platinum plus doxorubicin (adriamycin) plus cyclophosphamide; PC, platinum plus cyclophosphamide; PAI, platinum plus doxorubicin (adriamycin) plus ifosfamide, platinum plus doxorubicin (adriamycin).

**Table 2:** Salvage therapies in recurrent/progression patients (*N* = 98).

Variable	Total (*n* = 98)		Isolated (*n* = 14)		Disseminated (*n* = 84)		*p*-Value ^1^
	*n*	%	*n*	%	*n*	%	
Salvage treatment (First-line after primary treatment failed) ^2^							0.002
Supportive care	34	(36.2)	1	(7.7)	33	(40.7)	
CT alone +/− HT or target therapy	22	(23.4)	1	(7.7)	21	(25.9)	
RT alone +/− HT or target therapy	9	(9.6)	4	(30.8)	5	(6.2)	
Surgery +/− CT or RT or CRT or target	13	(13.8)	2	(15.4)	11	(13.6)	
CRT +/− HT or target	9	(9.6)	2	(15.4)	7	(8.6)	
HT alone	7	(7.4)	3	(23.1)	4	(4.9)	
Salvage chemotherapy regimen (First-line after primary treatment failed)							0.930
Not receive chemotherapy	58	(61.7)	9	(69.2)	49	(60.5)	
Paclitaxel +/− carboplatin/cisplatin	10	(10.6)	2	(15.4)	8	(9.9)	
Ifosfamide +/− carboplatin/cisplatin	2	(2.1)	0	(0)	2	(2.5)	
Doxorubicin/Lipodoxorubicin +/− carboplatin/cisplatin/Ifosfamide	7	(7.4)	1	(7.7)	6	(7.4)	
Platinum only	7	(7.4)	0	(0)	7	(8.6)	
Others	10	(10.6)	1	(7.7)	9	(11.1)	
Salvage target and immunotherapy (First-line after primary treatment failed)							0.436
Did not receive target/immunotherapy	85	(90.4)	12	(92.3)	73	(90.1)	
Pazopanib	4	(4.3)	0	(0)	4	(4.9)	
Bevacizumab	2	(2.1)	0	(0)	2	(2.5)	
Olaparib	1	(1.1)	1	(7.7)	0	(0)	
Pembrolizumab	1	(1.1)	0	(0)	1	(1.2)	
Bevacizumab + Pembrolizumab	1	(1.1)	0	(0)	1	(1.2)	

^1^ Comparing isolated and disseminated recurrence using Fisher’s Exact Test for categorical variables. ^2^ Salvage treatments of four patients were unknown. Abbreviations: CT, chemotherapy; CRT, chemoradiotherapy; HT, hormone therapy; RT, radiotherapy.

**Table 3:** Univariable and multivariate analyses of cancer-specific survival in recurrent/progressive uterine carcinosarcoma patients (*N* = 98).

		Univariate Analysis		Multivariate Analysis ^1^	
Variable	*n*	HR (95% CI)	*p*-Value	HR (95% CI)	*p*-Value
Age					
≤60 years	48	Ref			
>60 years	50	1.29 (0.84, 1.99)	0.239		
Stage					
I-II	26	Ref			
III-IV	72	1.42 (0.87, 2.31)	0.163		
CA-125 at recurrence					
≤35	34	Ref			
>35	48	1.13 (0.70, 1.82)	0.617		
Estrogen receptor					
Negative	49	Ref			
Positive	28	0.87 (0.53, 1.43)	0.576		
Progesterone receptor					
Negative	51	Ref			
Positive	26	0.76 (0.46, 1.26)	0.284		
Adjuvant treatment					
No	18	Ref			
Radiotherapy	7	1.48 (0.60, 3.62)	0.394		
Chemoradiotherapy	40	0.57 (0.31, 1.03)	0.060		
Chemotherapy	32	0.94 (0.51, 1.72)	0.843		
Hormone therapy	1	1.45 (0.19, 11.10)	0.720		
Time to treatment failure					
≤6 months	31	Ref		Ref	
>6 months	67	0.23 (0.14, 0.36)	0.000	0.30 (0.18, 0.52)	<0.001
Salvage treatment (first-line after primary treatment failed)					
Supportive care	34	Ref		Ref	
CT alone +/− HT or target	22	0.24 (0.13, 0.44)	0.000	0.29 (0.15, 0.53)	<0.001
RT alone +/− HT or target	9	0.11 (0.04, 0.31)	0.000	0.26 (0.10, 0.72)	0.010
Surgery +/- CT/RT/CRT or target	13	0.24 (0.11, 0.50)	0.000	0.44 (0.21, 0.93)	0.033
CRT +/− HT or target	9	0.23 (0.10, 0.53)	0.000	0.17 (0.07, 0.41)	<0.001
HT alone	7	0.16 (0.05, 0.46)	0.001	0.34 (0.11, 1.01)	0.053
Recurrence in previous RT field					
No	77	Ref			
Yes (infield failure)	21	0.91 (0.53, 1.56)	0.739		
Recurrent/progressive disease pattern					
Isolated	14	Ref		Ref	
Disseminated	84	4.82 (2.27, 10.25)	0.000	4.01 (1.55, 10.33)	0.004

^1^ Multivariate analysis excluded CA-125 at recurrence, ER, and PR because of missing data, which are substantial for these variables.

**Table 4:** Univariable and multivariate analysis of survival after recurrence in recurrent/progressive uterine carcinosarcoma patients (*N* = 98).

		Univariate Analysis		Multivariate Analysis ^1^	
Variable	*n*	HR (95% CI)	*p*-Value	HR (95% CI)	*p*-Value
Age					
≤60 years	48	Ref			
>60 years	50	1.06 (0.70, 1.63)	0.773		
Stage					
I-II	26	Ref			
III-IV	72	1.39 (0.86, 2.27)	0.182		
CA-125 at recurrence					
≤35	34	Ref			
>35	48	1.28 (0.79, 2.06)	0.316		
Estrogen receptor					
Negative	49	Ref			
Positive	28	0.99 (0.60, 1.62)	0.957		
Progesterone receptor					
Negative	51	Ref			
Positive	26	0.76 (0.46, 1.26)	0.287		
Time to treatment failure					
≤6 months	31	Ref			
>6 months	67	0.52 (0.33, 0.81)	0.004		
Salvage treatment (first-line after primary treatment failed)					
Supportive care	34	Ref		Ref	
CT alone +/− HT or target	22	0.41 (0.23, 0.71)	0.001	0.41 (0.24, 0.72)	0.002
RT alone +/− HT or target	9	0.15 (0.06, 0.39)	<0.001	0.27 (0.10, 0.71)	0.008
Surgery +/− CT/RT/CRT or target	13	0.35 (0.17, 0.73)	0.005	0.52 (0.25, 1.09)	0.085
CRT +/− HT or target	9	0.28 (0.13, 0.61)	0.002	0.33 (0.15, 0.75)	0.008
HT alone	7	0.28 (0.11, 0.72)	0.009	0.52 (0.19, 1.41)	0.201
Recurrence in previous RT field					
No	77	Ref			
Yes (infield failure)	21	0.86 (0.51, 1.45)	0.581		
Recurrent/progressive disease pattern					
Isolated	14	Ref		Ref	
Disseminated	84	4.58 (2.22, 9.45)	0.000	3.94 (1.67, 9.31)	0.002

^1^ Multivariate analysis excluded CA-125 at recurrence, ER, and PR because of missing data, which are substantial for these variables.

**Table 5:** Clinical features of long-term survivors (survival after recurrence > 50 months) in recurrent/progressive uterine carcinosarcoma patients.

No.	Age	FIGO Stage	Primary Treatment	CA125 ^2^	TTF	Site of Recurrence/Pattern	Salvage Therapy	SAR	CSS	Status
1	55.6	IA	Comprehensive surgical staging ^1^	3.99	2.50	Lung, pelvis, and vagina/disseminated pattern	CRT to pelvis and lung (ifosfamide + cisplatin)	168.2	170.7	DWOD (colon cancer)
2	52.4	II	CPSS + CT (cisplatin + paclitaxel)	6.0	19.02	Lung/solitary pattern	VATS resection of lung tumor	144.7+	163.7	NED
3	34.6	IIIB	CPSS + CRT (ifosfamide + cisplatin)	99.7	80.3	Left pelvic recurrence, extended to int. and ext. iliac LN and caused hydronephrosis (infield failure)/solitary pattern	Hormone therapy (anastrozole and megestrol acetate for ER3+, PR3+)	101.4	181.7	DOD
4	49.9	IIIC2	CPSS + CT (PAC)	NA	8.2	Neck LN/solitary pattern	RT to neck LN	88.0	96.2	DWOD (unknown)
5	68.4	IA	CPSS + CT (cisplatin + paclitaxel)	6.2	16.9	Vagina/solitary pattern	Vaginectomy and vaginal stump RT	79.3+	96.2	NED
6	65.7	IIIA	CPSS + CRT (ifosfamide+ doxorubicin)	9.5	15.5	Lung/solitary pattern	RT to lung nodule	53.9+	69.4	NED

^1^ Comprehensive surgical staging contained hysterectomy plus bilateral salpingo-oophrectomy plus pelvic lymph node dissection +/− paraaortic lymph dissection and omentectomy. ^2^ CA125 at first recurrence. Abbreviations: CPSS: comprehensive surgical staging; CT: chemotherapy; RT: radiotherapy; CRT: chemoradiation; CSS: cancer-specific survival; DWOD: died with other disease; DOD: died of disease; LN: lymph node; NED: no evidence of disease; PAC: cisplatin, doxorubicin and cyclophosphamide; SAR: survival after recurrence; TTF: time to treatment failure; VATS: video-assisted thoracoscopic surgery.

**Table 6:** Salvage treatment with target agents at first salvage attempt in recurrent/progressive uterine carcinosarcoma patients.

No	Age	FIGO Stage	Primary Treatment	IHC Markers	TTF	Site of Recurrence/Pattern	Salvage Therapy	SAR	CSS	Status
1	62.4	IVB	NACT + IDS + post-operative CRT (lipodox + ifoasfamide)	ER (−) PR(−) P53(+) MMR-p PDL1(+) BRCA1 somatic deletion	17.4	1st rec: Left inguinal LN (solitary pattern) 2nd rec: Right pelvic LN, ext. iliac	(1) RT to inguinal LN → CR maintenance with Olaparib (2) Pembrolizumab	14.7	32.0	DOD
2	61.9	IA	Comprehensive surgical staging (malignant ascites) + CT (TP)	ER (−) PR(−) P53(+) MMR-p PDL1 (−)	20.4	1st rec: Peritoneal carcinomatosis (disseminated pattern) 2nd rec: progressive with ometum cake and subdiaphragm seeding	(1) Cisplatin, bevacizumab, pembrolizumab →oral cyclophosphamide, tamoxifen, lenvatinib (2) RT to lower pelvis + TPB + oral palbociclib	21.4	41.8	DOD
3	69.0	IIIA	Hysterectomy at another hospital, refused adjuvant therapy.	ER (−) PR(+) P53(+) ^1^	3.7	Peritoneal carcinomatosis, bone metastasis /disseminated pattern	RT to pelvic tumor, with Cisplatin, bevacizumab → 5-FU + bevazumab	2.9	6.6	DOD
4	77.4	IVB	CRT (TPB)	ER (−) PR(−) P53(−) MMR-p	3.5	1st rec: Pelvic tumor, supraclavicular and axillary LN (disseminated pattern) 2nd rec: Progressive disease	(1) CT with Bevacizumab + oral tegafur, etoposide (2) paclitaxel + bevacizumab, Topotecan	6.4	9.9	DOD
5	37.1	IVB	Comprehensive surgical staging + CT (TP)	ER (−) PR(−) ^1^	7.1	Peritoneal carcinomatosis, neck and axillary LN, PALN, skin and breast nodule (disseminated pattern)	Lipodox+ Pembrolizumab → TP	2.2	9.3	DOD
6	70.4	IB	Comprehensive surgical staging + CRT (TP)	ER (−) PR(−) P53(−) MMR-d PDL1 (−)	16.0	1st rec: Lung and brain /disseminated pattern 2nd rec: New lung nodules	(1) RT to brain and lung, with CT AI, then maintenance with Pazopanib (2) Pembrolizumab	13.1	29.1	DOD
7	61.0	IIIA	Comprehensive surgical staging + CRT (TP)	ER (+) PR(+) P53(+) MMR-p	13.4	Malignant pleural effusion /disseminated pattern	TP, then maintained on oral pazopanib + cyclophosphamide	10.3	23.7	DOD
8	50.3	IIIC2	Comprehensive surgical staging + CRT (TP)	ER (−) PR(−) P53(+) MMR-p	12.9	Lung and bone/disseminated pattern	VATS lobectomy for lung metastasis, then oral pazopanib	2.0	14.8	DOD
9	48.4	IVB	Comprehensive surgical staging + CRT (TP)	ER (+) PR(+) MMR-p PDL1 (−)	5.9	Peritoneal carcinomatosis, liver metastasis/disseminated pattern	RT to liver, oral letrozole, megace, and pazopanib	6.3	12.3	DOD

^1^ This patient could not undergo more IHC testing due to lack of specimen. Abbreviations: AI: adriamycin(doxorubicin) plus ifosfamide; CR: complete remission; LN: lymph node; Rec: recurrence; NACT plus IDS: neoadjuvant chemotherapy plus interval debulking surgery; IHC: immunohistochemistry staining; MMR-p: mismatch repair-proficient; MMR-d: mismatch repair-deficient; RT: radiotherapy; TTF: time to treatment failure; VATS: video-assisted thoracoscopic surgery; TPB: paclitaxel plus platinum plus bevacizumab; TP: paclitaxel plus platinum.

## Data Availability

The data presented in this study are available on request from the corresponding author.

## Supplementary Materials

The following supporting information can be downloaded at: https://www.mdpi.com/article/10.3390/curroncol29100601/s1. Figure S1: Flow diagram of UCS patients; Figure S2: Survival analysis of SAR stratified by recurrent patterns; Figure S3A: Survival analysis of SAR stratified by salvage treatments in stage I and II patients; Figure S3B: Survival analysis of SAR stratified by salvage treatments in stage III and IV patients; Figure S4: SAR of patients received salvage chemotherapy regimen containing ifosfamide; Figure S5. SAR of patients received salvage chemotherapy regimen chemotherapy containing paclitaxel or platinum; Figure S6. SAR of patients received salvage target/immune therapy; Table S1. Univariable and multivariate analysis of survival after recurrence in disseminated patients (*N* = 84); Table S2. Kaplan-Meier analysis of survival after recurrence in isolated recurrent patients (*N* = 14).
