# Supplementary material for: Management and Prognosis of Patients with Recurrent or Persistent/Progressive Uterine Carcinosarcoma
Source: Curr Oncol. 2022 Oct 13;29(10):7607–23. doi: 10.3390/curroncol29100601 (PMC9600185; doi:10.3390/curroncol29100601)
Supplement: Supplementary file 1 [file curroncol-29-00601-s001.zip › curroncol-1893176-supplementary.pdf]

Supplementary Figure S1. Flow diagram of UCS patients

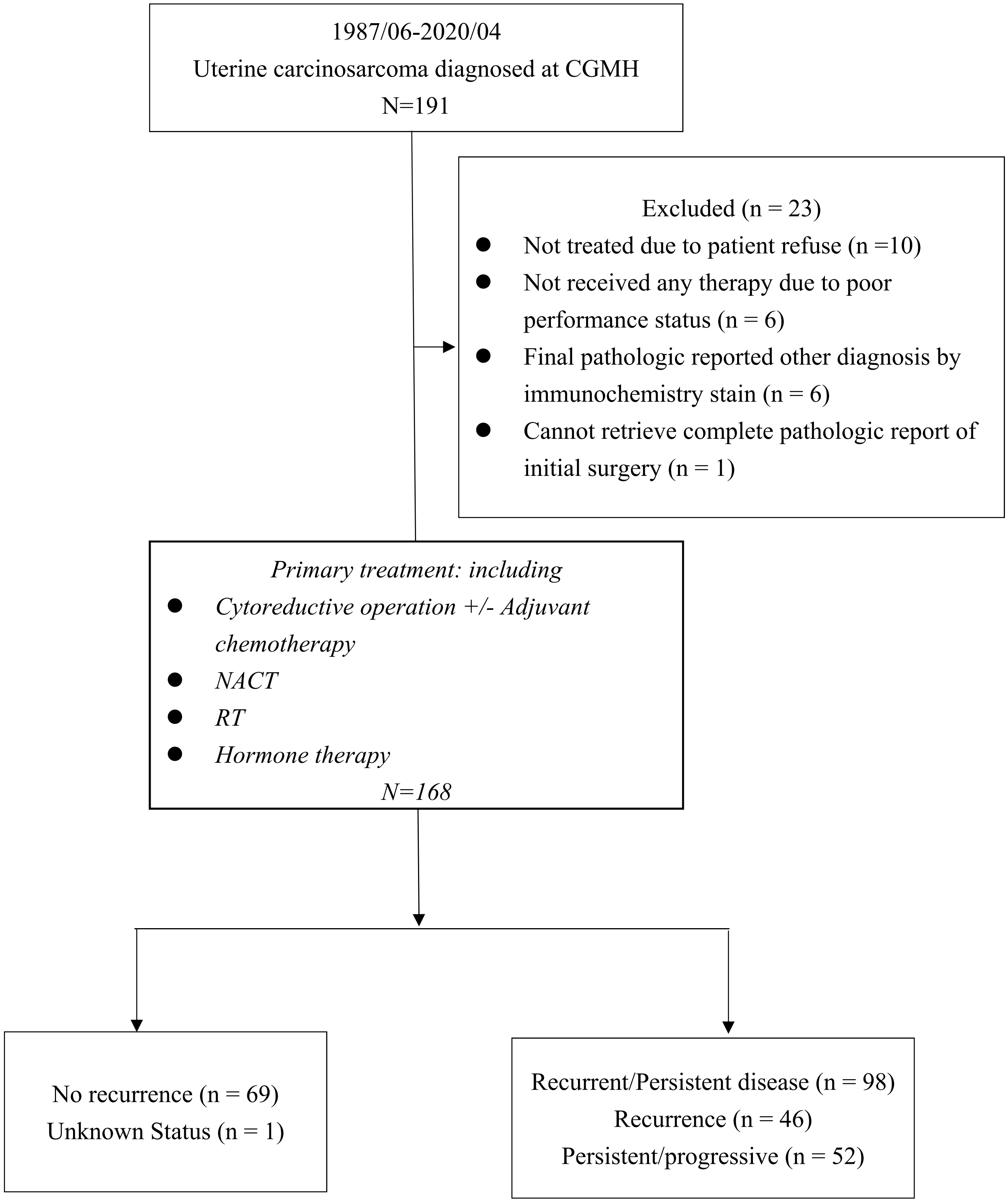

Supplementary Figure S2. Survival analysis of SAR stratified by recurrent patterns

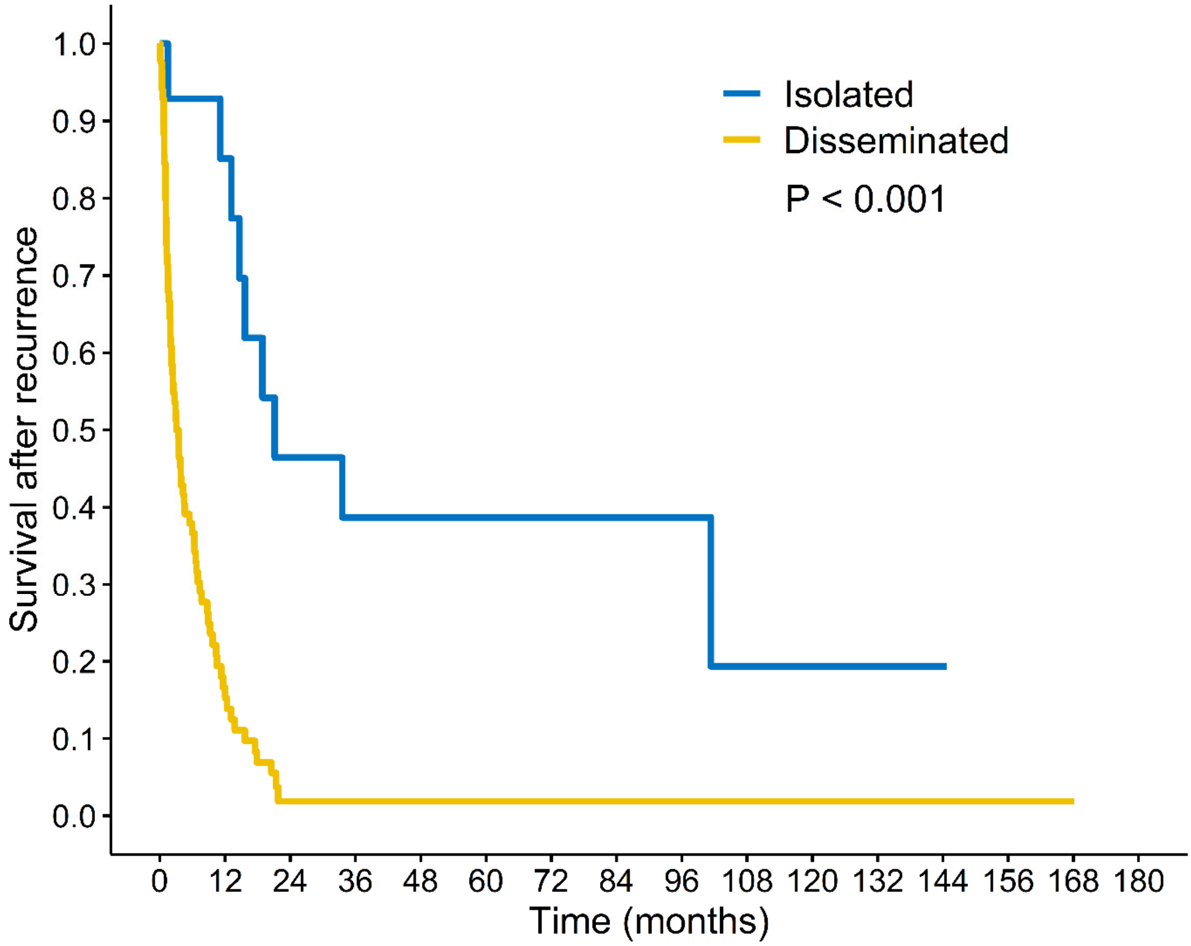

Number at risk

Isolated	14	11	6	5	5	4	4	3	2	1	1	1	1	0	0	0
Disseminated	84	12	1	1	1	1	1	1	1	1	1	1	1	1	1	0

Supplementary Figure S3A. Survival analysis of SAR stratified by salvage treatments in stage I and II patients

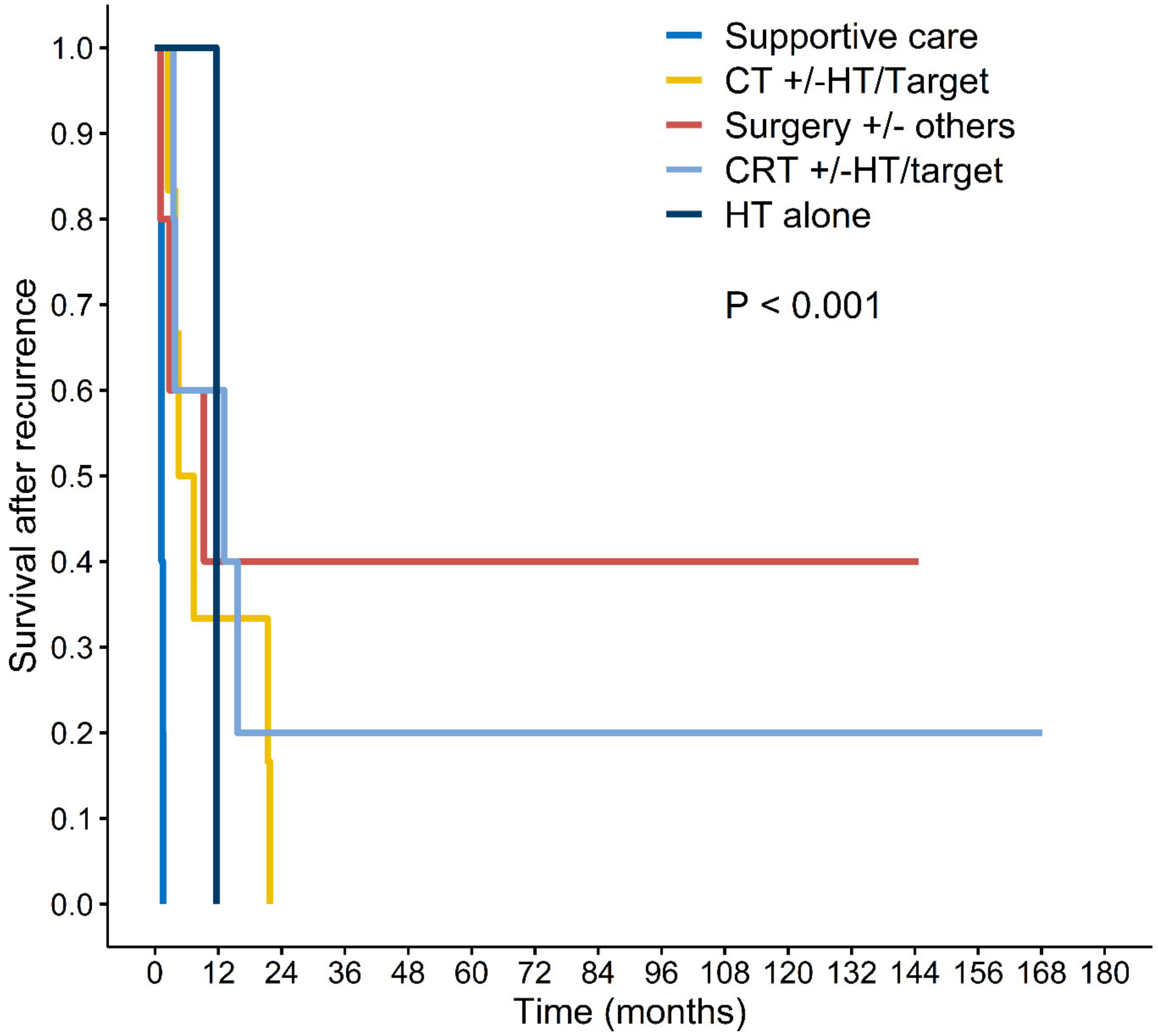

Number at risk

	0	12	24	36	48	60	72	84	96	108	120	132	144	156	168	180
Supportive care	5	0	0	0	0	0	0	0	0	0	0	0	0	0	0	0
CT +/-HT/Target	6	2	0	0	0	0	0	0	0	0	0	0	0	0	0	0
Surgery +/- others	5	2	2	2	2	2	2	1	1	1	1	1	1	0	0	0
CRT +/-HT/target	5	3	1	1	1	1	1	1	1	1	1	1	1	1	1	0
HT alone	2	0	0	0	0	0	0	0	0	0	0	0	0	0	0	0

Supplementary Figure S3B. Survival analysis of SAR stratified by salvage treatments in stage III and IV patients

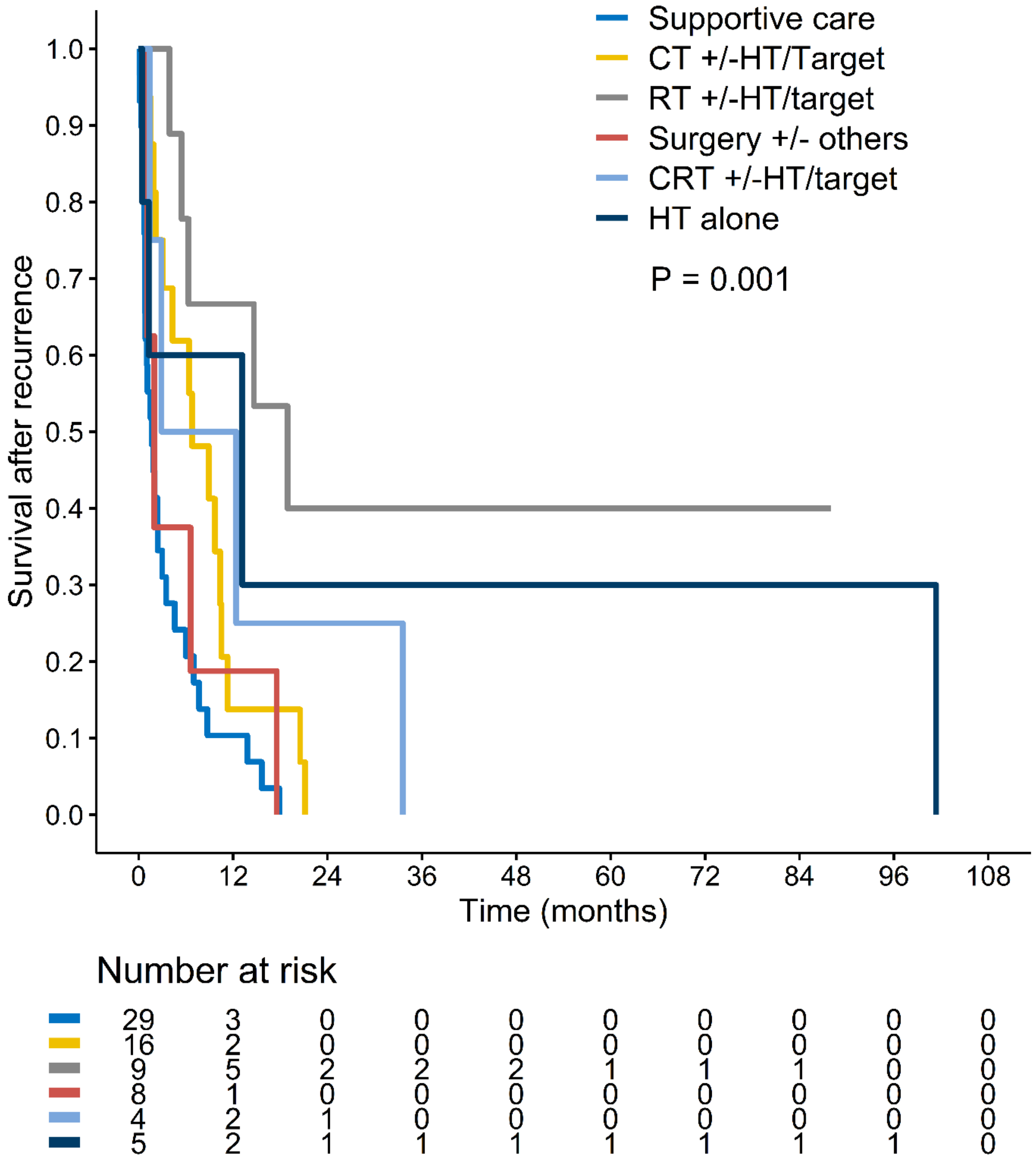

Supplementary Figure S4. SAR of patients received salvage chemotherapy regimen containing ifosfamide.

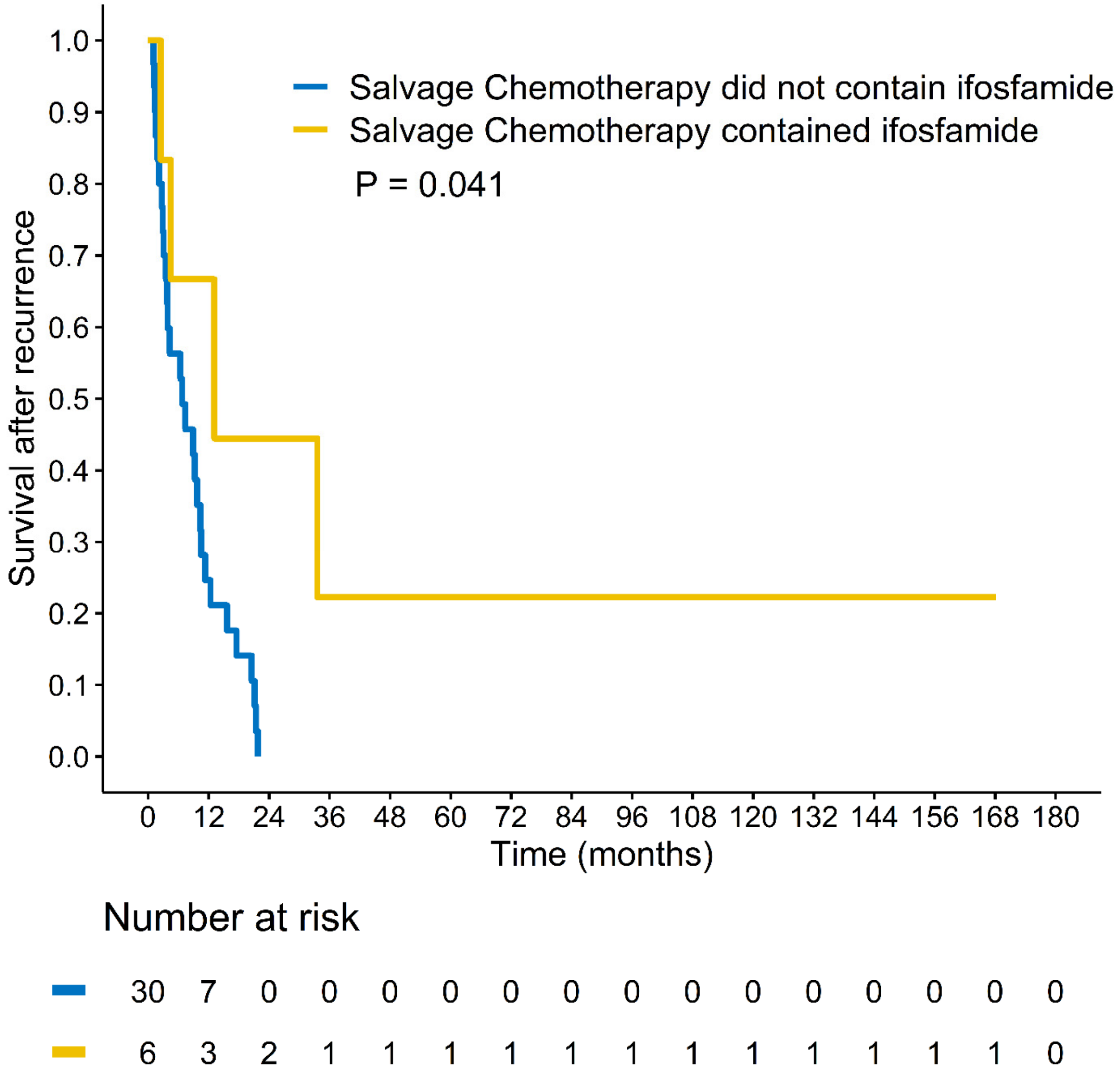

Supplementary Figure S5. SAR of patients received salvage chemotherapy regimen chemotherapy containing paclitaxel or platinum.

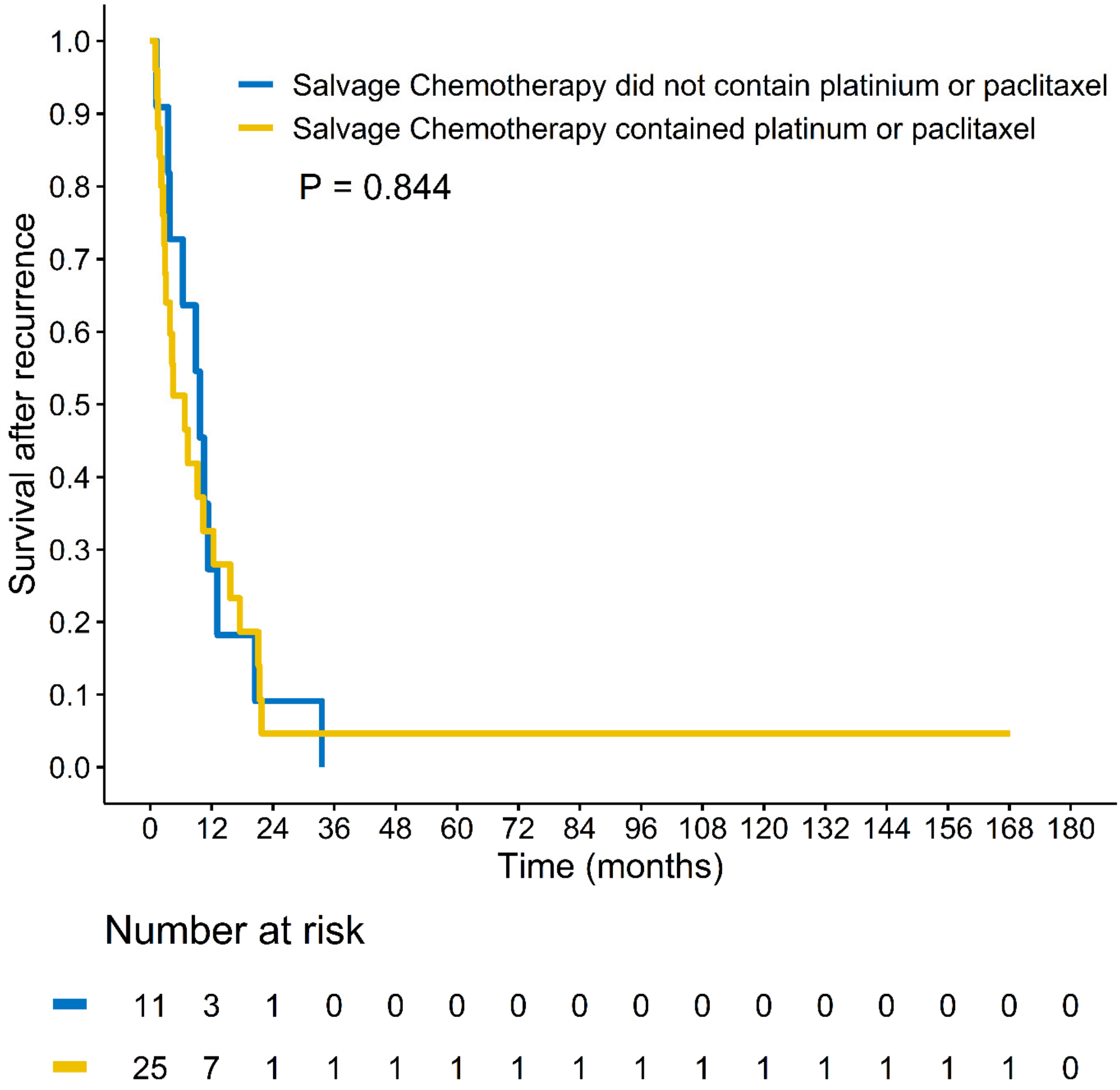

Supplementary Figure S6. SAR of patients received salvage target/immune therapy.

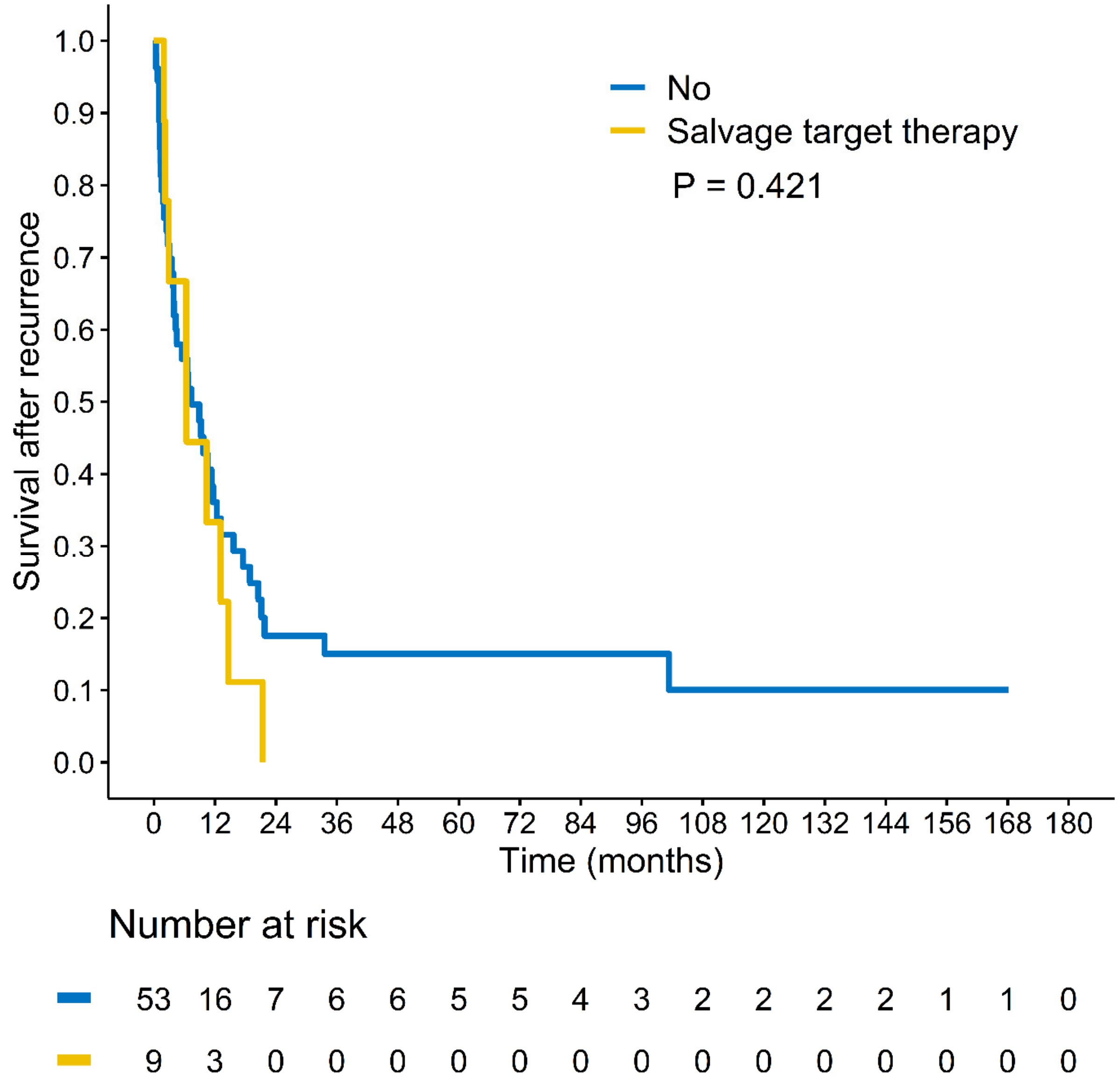

Supplementary Table S1. Univariable and multivariate analysis of survival after recurrence in disseminated patients (N=84)

Variable	n	Univariate analysis		Multivariate analysis[1]	
		HR (95% CI)	p-value	HR (95% CI)	p-value
Age					
≤60 year-old	41	Ref			
>60 year-old	43	0.89 (0.57, 1.39)	0.612		
Stage					
I-II	21	Ref			
III-IV	63	1.53 (0.89, 2.64)	0.123		
CA-125 at recurrence					
≤ 35	27	Ref			
> 35	43	1.05 (0.63, 1.75)	0.846		
Estrogen receptor					
Negative	39	Ref			
Positive	25	0.93 (0.58, 1.65)	0.926		
Progesterone receptor					
Negative	43	Ref			
Positive	21	0.82 (0.48, 1.41)	0.482		
Time to treatment failure					
≤ 6months	31	Ref			
> 6months	53	0.71 (0.45, 1.12)	0.139		
Salvage treatment (1st-line after primary treatment failed)					
Supportive care	33	Ref		Ref	
CT alone +/- HT or target	21	0.42 (0.23, 0.74)	0.003	0.42 (0.23, 0.74)	0.003
RT alone +/- HT or target	5	0.26 (0.08, 0.84)	0.024	0.26 (0.08, 0.84)	0.024
Surgery +/- CT/RT/CRT or Target	11	0.71 (0.35, 1.45)	0.352	0.71 (0.35, 1.45)	0.352
CRT +/- HT or target	7	0.33 (0.13, 0.81)	0.015	0.33 (0.13, 0.81)	0.015
HT	4	0.50 (0.15, 1.66)	0.259	0.50 (0.15, 1.66)	0.259
Recurrence in previous RT field					
No	65	Ref			
Yes (Infield failure)	19	0.76 (0.44, 1.33)	0.343		

[1]Multivariate analysis excluded CA-125 at recurrence, ER, and PR, because of missing data.
Abbreviations: CT, chemotherapy; CRT, chemoradiotherapy; HT, hormone therapy; RT, radiotherapy

Supplementary Table S2. Kaplan-Meier analysis of survival after recurrence in isolated recurrent patients (N=14)

Variable	n	Median , (range) months	3-year SAR	5-year SAR	p-value
Age[1]					
≤70 year-old	11	33.6 (3.4, 144.7)	50.0%	50.0%	<0.001
>70 year-old	3	11.1 (1.5, 13.2)	0	0	
Stage					
I-II	5	15.6 (1.5, 144.7)	40.0%	40.0%	0.980
III-IV	9	10.4 (3.4, 101.4)	37.5%	37.5%	
CA-125 at recurrence					
≤ 35	7	18.9 (1.5, 144.7)	42.9%	42.9%	0.701
> 35	5	21.2 (3.4, 101.4)	25.0%	25.0%	
Estrogen receptor					
Negative	10	18.9 (1.5, 88.0)	33.8%	33.8%	0.245
Positive	3	101.4 (33.6, 144.7)	66.7%	66.7%	
Progesterone receptor					
Negative	8	14.7 (1.5, 88.0)	43.8%	43.8%	0.651
Positive	5	33.6 (18.9, 144.7)	40.0%	40.0%	
Time to treatment failure[2]					
≤ 12months	5	15.6 (1.5, 88.0)	20.0%	20.0%	0.239
> 12months	9	21.2 (3.4, 144.7)	50.0%	50.0%	
Salvage treatment with CT alone+/- HT or target					
Yes	1	21.2 (21.2, 21.2)	0	0	0.732
No	13	33.6 (1.5, 144.7)	42.0%	42.0%	
Salvage treatment with RT alone+/- HT or target					
Yes	4	18.9 (14.7, 88.0)	50.0%	50.0%	0.595
No	10	21.2 (1.5, 144.7)	33.8%	33.8%	
Salvage treatment with CRT alone+/- HT or target					
Yes	2	15.6 (15.6, 33.6)	0	0	0.537
No	12	21.2 (1.5, 144.7)	45.8%	45.8%	
Salvage treatment with Surgery alone+/- HT or target					
Yes	2	79.3 (79.3, 144.7)	100%	100%	0.072
No	12	18.9 (1.5, 101.4)	27.5%	27.5%	
Salvage treatment with HT alone					
Yes	3	13.2 (3.4, 101.4)	50.0%	50.0%	0.795

No	11	21.2 (1.5, 144.7)	36.4%	36.4%	
Recurrence in previous RT field					
No	12	18.9 (1.5, 144.7)	36.7%	36.7%	0.925
Yes (Infield failure)	2	21.2 (21.2, 101.4)	50.0%	50.0%	

[1]Age cut-offs at 65, 60, and 55 year-old were not significant.

[2]TTF cut-offs at 6, 18 months were not significant.

Abbreviations: CT, chemotherapy; CRT, chemoradiotherapy; HT, hormone therapy; RT, radiotherapy
